# New Insights Into Implementation of Mesenchymal Stem Cells in Cancer Therapy: Prospects for Anti-angiogenesis Treatment

**DOI:** 10.3389/fonc.2019.00840

**Published:** 2019-08-28

**Authors:** Mohammad Reza Javan, Arezou Khosrojerdi, Seyed Mohammad Moazzeni

**Affiliations:** Department of Immunology, Faculty of Medical Sciences, Tarbiat Modares University, Tehran, Iran

**Keywords:** angiogenesis, mesenchymal stem cells, tumor microenvironment, cancer therapy, drug delivery

## Abstract

Tumor microenvironment interacts with tumor cells, establishing an atmosphere to contribute or suppress the tumor development. Among the cells which play a role in the tumor microenvironment, mesenchymal stem cells (MSCs) have been demonstrated to possess the ability to orchestrate the fate of tumor cells, drawing the attention to the field. MSCs have been considered as cells with double-bladed effects, implicating either tumorigenic or anti-tumor activity. On the other side, the promising potential of MSCs in treating human cancer cells has been observed from the clinical studies. Among the beneficial characteristics of MSCs is the natural tumor-trophic migration ability, providing facility for drug delivery and, therefore, targeted treatment to detach tumor and metastatic cells. Moreover, these cells have been the target of engineering approaches, due to their easily implemented traits, in order to obtain the desired expression of anti-angiogenic, anti-proliferative, and pro-apoptotic properties, according to the tumor type. Tumor angiogenesis is the key characteristic of tumor progression and metastasis. Manipulation of angiogenesis has become an attractive approach for cancer therapy since the introduction of the first angiogenesis inhibitor, namely bevacizumab, for metastatic colorectal cancer therapy. This review tries to conclude the approaches, with focus on anti-angiogenesis approach, in implementing the MSCs to combat against tumor cell progression.

## Introduction

Cancer is considered as one of the top life-threatening complications, which is responsible for a quarter of human mortalities (1). The common cancer therapies, such as surgery, chemotherapy and radiotherapy, are usually symptomatic and passive in their procedures. In spite of improved treatment approaches, most of the cancer cells do not respond properly to traditional therapeutics. The major hindrance in limiting the efficacy of traditional cancer therapeutics is tumor specificity. As a consequence, it is important to look for efficient therapeutic strategies specifically targeting malignancies (2).

MSCs are the first choice of stem cells for application in clinical medicine. A number of these cells have been extracted from different tissues, such as the heart, brain, and kidney, and have demonstrated the potential as attractive candidates for treating various types of diseases (3, 4). The potential of MSC to differentiate into various cell types, and the simplicity of expansion *in vitro*, have raised the interests toward their therapeutic applications to treat human diseases. They can be extracted from adult human tissues and have the ability for self-renewal and development into mesenchymal lineages. They are re-grafted following proliferation and manipulation *in vitro*. After re-implantation, they gain the potential to suppress the immune system. Furthermore, they can be adapted into the re-grafted tissue architecture and promoted to progeny with regard to both stem cells and lineage of daughters in the microenvironment. MSCs show pathotropic migratory characteristics, providing them as potential tools for application in targeted delivery vectors for tumor therapy (3, 4). With the advent of specific anticancer drugs and considering the capacity of MSCs in tumor-targeted delivery and incorporation, a new research area has emerged with the aim of developing efficient cancer therapy using manipulated MSCs.

Tumor angiogenesis is an approach for tumor cells for their proliferation (5). The observations that several growth factors and mediators of the extracellular matrix account for tumor angiogenesis raised attention for the utilization of targeted anti-angiogenic therapy of cancers (6). On the other side, different types of stem cells have been modulated to express anti-angiogenic factors (3).

In this review, after general characterization of MSCs, we go through the approaches for application of MSCs in treatment of cancers. Alternately, with respect to the possible anti-angiogenic properties of MSCs in normal human physiology, we proposed a putative straightforward application of these cells in cancer therapy.

## Characteristics of MSCs

MSCs are a group of adult stem cells, which are naturally developed in the human body. They were first identified in the stromal matrix of bone marrow by Friedenstein et al. (7, 8). The comprehensive nature and localization of MSCs *in vivo* remain poorly known. Other than bone marrow, MSCs have been found in a number of other adult and fetal tissues, such as heart, amniotic fluid, skeletal muscle, synovial tissue, adipose tissue, pancreas, placenta, cord blood and circulating blood. It has been suggested that basically all organs containing connective tissue possess MSCs (9). Among the stem cells, MSCs are the most investigated and the best-defined stem cells. MSCs are primitive cells, which originate from the mesodermal germ layer and were classically known as progenitors developing to connective tissues, skeletal muscle cells, and cells of the vascular system. MSCs can develop into cells of the mesodermal lineage, like bone, fat and cartilage cells, but they have the potential to differentiate into endodermic and neuroectodermic lineages. In fact, bone marrow-derived MSCs are a heterogeneous population (10). Because of their supposed capacity of self-renewal and differentiation, bone marrow-derived stromal cells were first regarded as stem cells and named MSCs (11), despite some controversy regarding their nomenclature (12). MSCs have emerged as considerable biomedical sources as a result of their multilineage potential (13). Due to their easy acquisition, fast *ex vivo* proliferation and the feasibility of autologous transplantation, MSCs became the first choice of stem cells to be applied in the clinical regenerative medicine. They may provide important potentials for cell survival in injured tissues, with or without direct participation in long-term tissue repairmen procedures (14). MSCs can modify the response of immune cells and therefore are linked with immune-related disorders, especially autoimmune settings (15, 16). MSCs have been shown to have specific tumor-oriented migration as well as incorporation capacity in several preclinical models, demonstrating the potential for MSCs to be used as favorable carriers for anticancer compounds (17). Bone marrow-derived MSCs obtained from other tissues, like adipose tissue, can also be potentially utilized as anticancer gene vehicles for cancer treatment (18, 19). MSCs show both pro- and anti-cancer features (20), providing “double-edged sword” characteristics in their interaction with tumor cells. However, if MSCs are suitably manipulated with anticancer genes they could be used as a favorable “single-edged sword” against cancer cells.

## Origin of MSCs

MSCs can be extracted from adult human tissues and have the potential for self-renewal and differentiation into mesenchymal lineages, such as chondrocytic, osteocytic, and adipogenic. The harvesting of MSC generally does not comply with ethical issues and is less invasive than other sources, for example neural stem cells (3). MSCs have the potential to develop into tissue types of other lineages, both within or across germ lines (21). The highest degree of lineage plasticity has been implicated in bone marrow-derived MSCs, which are capable of giving rise to virtually all cell types upon implantation into early blastocysts and are relatively easy to manipulate *in vitro* (22, 23). To date, most of the preclinical studies have been done with bone marrow-derived MSCs, which might not be the best-suited source available for the clinical applications. The harvesting of bone marrow requires invasive steps which yields a small number of cells, and the number, differentiation potential, and life span of bone marrow-derived MSCs reduces alongside with the age of the patient (24, 25). Two other accessory sources for harvesting MSCs that have received significant attention are adipose tissue and umbilical cord blood. MSCs derived from adipose have become a highly attractive alternative in recent years, mainly due to the ease of tissue collection, high initial cell yields, and favorable proliferation ability *in vitro* (26). The expansion and differentiation capacity as well as the immunophenotype of MSCs obtained from adipose tissue are nearly the same as those extracted from bone marrow (27). Immunogenicity of allogeneic and xenogeneic MSCs isolated from adipose tissue has been shown not to be a problematic issue for their therapeutic applications, at least in recurrent spontaneous abortion (28). Moreover, MSC therapy could modulate the immune responses in a beneficial way (29). In fact, MSC therapy modulated the balance of helper T (Th) 1/Th2 cytokines production toward increased Th2 type cytokines (30).

Umbilical cord blood and Wharton's jelly have been shown to be a rich source of MSCs (31). As the cells are isolated after removal of the placenta, the extraction procedure is completely non-invasive and simple. The cells in the adherent layer have been demonstrated to have a fibroblastiod morphology, which express the same surface markers as bone marrow-derived MSCs, including CD13, CD29, CD49e, CD54, and CD90 (32). MSCs with umbilical cord blood origin have the potential to expand at a higher speed in relation to bone marrow and adipose tissue-derived MSCs (27, 33). This issue may be somewhat due to higher telomerase activity (34).

Adult dental pulp (DP) is another source of MSC, which has been explored and validated. DP is a vascular connective tissue like mesenchymal tissue. Stem cells isolated from DP have a phenotype similar to the adult bone marrow-derived MSCs that express mesenchymal progenitor-related antigens, including SH2, SH3, SH4, CD166, and CD29. Moreover, the DP and bone marrow-derived stem cell populations show similar profiles of gene expression (35, 36). DP-derived MSCs are obtained from a very accessible tissue source, which is further expandable using deciduous teeth, that show stem cell-like properties, such as efficient multilineage differentiation and self-renewal. Furthermore, their capacity to stimulate osteogenesis could be beneficial in clinical use for implantology (37). DP-derived MSCs can show potential clinical applications in autologous *in vivo* stem cell transplantation in order to rebuild the calcified tissue. Immunomodulatory function of DP-derived MSCs is suitable for suppression of T cell-mediated responses in case of allogeneic transplantation of bone marrow (36, 37).

## Colonization and Migration of MSCs

**Figure 1:**
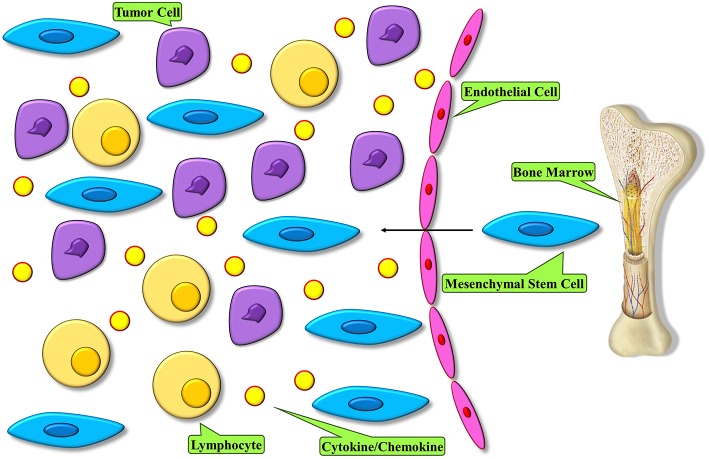
MSC homing within tumors. Tumor microenvironment modulates an inflammatory condition and releases mediators to recruit MSCs into tumor. These cells become the major constituent of the tumor microenvironment.

The very first key step for MSC homing to tumors is their mobilization from the bone marrow and other organs. Endogenous MSCs have been demonstrated to mobilize from the bone marrow and other tissues to the peripheral blood during different injury conditions, such as normoxia, hypoxia, and inflammatory conditions (38, 39) (Figure 1). The mechanisms underlying MSC migration across the endothelium and homing to the target tissues are not fully described. Nonetheless, it is known that a normal role of MSC is the potential to migrate to and repair wounded tissue. This feature of wound healing originates with migration toward inflammatory signals released by the wounded tissue (40). Several inflammatory mediators that are produced by wounds are found in the tumor microenvironment and are believed to be involved in attracting MSCs to these sites (41). Furthermore, we indicated that pro-inflammatory cytokines were able to upregulate the efficacy of MSCs in a cell-based therapy of various complications (42). A bulk of studies has demonstrated that migration is mediated through cytokines secreted by MSCs that are involved with G-protein coupled receptor (GPCR), growth factor receptors, and various cytokine/receptor pairs SDF-1/CXCR4, SCF-c-Kit, HGF/c- Met, VEGF/VEGFR, PDGF/PDGFR, HMGB1/RAGE, and MCP-1/CCR2 [N (43)]. Stromal cell-derived factor SDF-1 and its receptor, namely CXC chemokine receptor-4 (CXCR4), are major mediators of stem cell infiltration to the tumor microenvironment. The significance of the interactions between secreted SDF-1 and cell surface CXCR4 for stem cell migration has been displayed by studies in which the function of either the receptor or the cytokine was downregulated (44–46). CXCR4 and SDF-1 simultaneous blockade *in vivo* in mice resulted in significant reduction in the migration of transplanted stem cells to tumor locations and regions of demyelination, implicating that the SDF-1/CXCR4 signaling pathway is important for effective pathotropism quality of the stem cell therapy (44). Other studies with blockades of both CXCR4 and transforming growth factor (TGF)-β receptor have shown that CXCR4 is required for endogenous MSC colonization into tumors, differentiation to myofibroblasts, and survival of MSCs (47). MSCs express a wide range of chemokine receptors, such as CXCR1, CXCR3, CXCR4, CXCR5, CXCR6, chemokine receptor (CCR)-1, CCR2, CCR3, CCR4, CCR5, and CCR9, among others. Studies have shown that chemokines such as CXCL12, CXCL13, CXCL16, and their related receptors can promote the bidirectional migration of MSCs to and create the bone marrow niche. Specific chemokines and their receptors play a role in the unidirectional migration of MSCs. In effective homing of MSC into the bone marrow, CXCL16 plays vital role, whereas CCL22 has the strongest chemotactic effect in mobilizing MSCs from the bone marrow into the circulation (48). Both CCL2 and CXCL16 are expressed in tumor tissues, such as lung carcinoma, hepatocellular carcinoma, colorectal cancer, and brain and ovarian cancers, hence it is possible that they play a role in the migration of MSC into tumors (49–51). Transactivation of cell receptor is a mechanism which is thought to be involved in migration and plays a role in the activation of one receptor through another. While it plays an important role in processes like cellular migration and apoptosis, its deregulation can cause pathological settings like cancer. On the other side, transactivation of various growth factor receptors, such as epidermal growth factor receptor (EGFR) by GPCRs, has been observed in multiple cellular model systems, therefore illustrating the plausible role of GPCR in tumor tropism by means of receptor transactivation (52–54). A suggested mechanism of receptor transactivation involves the activation of membrane-tethered growth factors, like EGFR, by direct interaction with GPCR, like CXCR4 (55). This procedure is accompanied by matrix metalloproteinases (MMPs), like MMP-2 and MMP-9 (56). MMPs are enzymes with proteinases activity that are required to proteolytically process precursor proteins like growth factors, adhesion molecules, cytokines, and their receptors. Migration of MSCs through bone marrow endothelium has been reported to be mediated by MMP-1 as well as the tissue inhibitor of metalloproteinase-3 (57). Another report demonstrated that elevated levels of MMP-2 were responsible for C1q complement protein-mediated migration of umbilical cord blood-derived MSC into harmed tissue (58). Studies on MSC behavior show that MSCs are attracted to the sites of irradiation, and that local irradiation might enhance the specificity of MSCs to be migrated and engrafted (59). Taken together, these observations suggest a strong validation for the development of therapeutic approaches that employ the tumoritropic characteristics of MSCs by engineering them into delivery vehicles for antitumor compounds.

## MSC Behavior in Tumor Microenvironment

**Figure 2:**
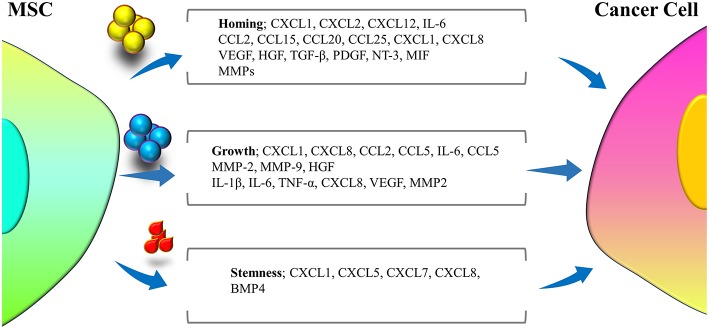
Different cytokines and mediators secreted from MSCs during cross-talk with tumor cells that are involved in three major interfaces of homing, growth, and stemness of tumor cells.

MSCs represent various functions in distinct microenvironments, through secreting several mediators (Figure 2), due to diversity of the signaling pathways that stimulate these cells. The MSCs applied for clinical treatment are predominantly naïve MSCs driven from normal tissue sources and are harvested *in vitro*. Naïve MSCs have the potential to interrupt tumor cell growth when co-cultured with tumor cells *in vitro*. A study demonstrated that naïve MSCs inhibited the proliferation of solid tumor and leukemia cell lines. The inhibitory impression of naïve MSCs was observed to be dose-dependent, as proportions of naïve MSCs were accompanied with higher levels of inhibition (60). The possible mechanism of tumor inhibition via naïve MSCs was attributed to the soluble factors secreted by these cells, including Dickkopf-related protein 1 (DKK1), which plays a role in the inhibition of Wnt signaling pathways in tumor cells. Interruption in the Wnt pathway downregulated Cyclin D2 and c-Myc and upmodulated expression of P27KIP1 and P21CIP1, leading to tumor cell cycle suppression (61–64). Moreover, naïve MSCs can also activate apoptosis in tumor cells (65) through upregulation of caspase 3 (61). Naïve MSCs, by inhibiting angiogenesis, can indirectly interrupt tumor expansion. The mechanisms underlying angiogenesis interruption by naïve MSCs are apoptosis in vascular endothelial cells (66, 67) and impairing the vascular network construction (68). Nonetheless, adverse roles of naïve MSCs have also been reported, as these cells were related to enhanced tumor angiogenesis in colon cancer cell lines (69, 70), MSC differentiation to vascular endothelial cells in melanoma (71), and promotion of cancer stem cells (CSCs) that enhance tumorigenesis, metastasis and recurrence of tumors (72) like breast cancer (73), increasing the expansion of gastric cancer cell lines (72, 74). Among the other unwanted influences of naïve MSCs in tumors are enhancement of tumor cell migration mediated by release of chemokines, such as CXCR4 (75), CCL5 (76, 77), vascular cell adhesion molecules (VCAMs), and intercellular adhesion molecules (ICAMs) (78). Additionally, MSCs were observed to suppress immune responses, leading to tumor development (79). Naïve MSCs have been associated with bidirectional effects on tumor growth and development, and different investigations have been accompanied by discrepancies on whether MSCs are involved in tumor promotion or suppression. The divergence in observed outcomes may stem from differences in experimental settings, in which different observations may be achieved by exerting *in vivo* or *in vitro* experiments.

The other type of MSCs, the tumor-derived MSCs (T-MSCs), demonstrate phenotype similarity with naïve MSCs but exhibit stronger immunosuppressive activity and proliferative capacity than naïve MSCs (80). MSCs isolated from mouse lymphomas (L-MSCs) compared with bone marrow-derived MSCs (BM-MSCs) promoted tumor cell proliferation vigorously, which was related to release of CCL2 by L-MSCs, resulting in recruitment of immunosuppressive cells, such as F4/80+ macrophages, CD11b+Ly6G+ neutrophils, and CD11b+ Ly6C+ monocytes to lymphoid tissues (81). Moreover, MSCs derived from breast cancer tissues relative to MSCs from normal breast tissues present higher amounts of immunosuppressive mediators such as TGF-β1, IL-4, and IL-10. Additionally, MSCs isolated from breast cancer tissues co-cultured with peripheral blood mononuclear cells (PBMCs) led to the development of CD4+CD25^hi^Foxp3+ regulatory T cells (82). Furthermore, MSCs derived from pediatric sarcomas demonstrated inhibitory effects of the cytotoxic functions of natural killer (NK) cells through downregulating the activation receptors of NK cells (83). On the other side, T-MSCs have also disadvantageous ramifications. T-MSCs exert a function in epithelial-to-mesenchymal transition (EMT), as pancreatic cancer revealed upregulation of vimentin and downregulation of E-cadherin (84). Moreover, MSCs from prostate cancer and breast cancer cell lines overexpress CCL5, which in turn up-modulated the expression of genes involved in EMT, such as zinc finger E-box-binding homeobox 1, CXCR4, and Snail (76, 85). As well, T-MSCs obtained from ovarian cancer increased the proliferation of tumor cells (86). Additionally, T-MSCs promoted the proportion of CSCs, as observed in ovarian cancer cell, probably because of upregulation of bone morphogenetic proteins (BMPs), such as BMP2, BMP4, and BMP6 in ovarian cancer-derived MSCs (86). Taken together, in contrast to naïve MSCs, T-MSCs demonstrate further capacities in promotion of tumor progression.

## The Potential of MSCs For Tumor Therapy

MSCs with no modification have been demonstrated to have antitumor effects both *in vitro* and in different mouse models of tumors. This is in part due to the factors released by MSC that have antitumor functions that can reduce the proliferation of melanoma, glioma, lung cancer, hepatoma, and breast adenocarcinoma (63, 87–89). BM-MSCs injected intravenously in a mouse model of Kaposi's sarcoma were shown to be colonized in sites of tumorigenesis and potently inhibit tumor development (90). MSCs have also been observed to have anti-angiogenic properties both *in vitro* and in mouse model of melanoma (66). Moreover, direct MSC injection into subcutaneous melanoma in mice triggered apoptosis and diminished tumor growth (66). Furthermore, umbilical cord blood-derived MSCs have been used as naïve cells for the therapy of glioblastoma multiforme (GBM). Umbilical cord blood-derived stem cells expressing high levels of CD44 and CD133 cells underwent apoptosis following co-culturing with GBM cells (91). Additionally, treatment of glioma cells with umbilical cord blood-derived stem cells caused inhibition of focal adhesion kinase (FAK)-mediated angiogenesis (92), upregulation of phosphatase and tensin homolog (PTEN) expression in glioma cells, and down-modulation of Akt and PI3K signaling pathway molecules in the nude mice tumors, resulting in the inhibition of migration as well as wound healing characteristics of the glioma cells (93). Alternately, this eventuated in downregulation of XIAP activating caspase-3 and caspase-9 to stimulate apoptosis in glioma cells (94). Another investigation surveyed a comparative study of umbilical cord blood-derived MSCs and MSCs from other sources and showed diminished GBM growth via tumor necrosis factor (TNF)-related apoptosis-inducing ligand (TRAIL) (95). In consideration of these observations, MSCs have been modified mainly to overexpress target genes in order to express or secrete a selective favorable therapeutic molecule for targeted treatment of various cancer types. These compounds mainly include interleukins, interferons, pro-apoptotic proteins, and anti-angiogenesis agents.

### Interleukins

Interleukins are cytokines that modulate immune responses and have been suggested to show antitumor properties through either direct tumoricidal effects or positive regulation of the endogenous immune system against tumor cells (96). Nonetheless, lack of tumor-targeted delivery has diminished their application for cancer treatment (97). With respect of results, the delivery of interleukins by MSCs has been investigated. Engineered MSCs for expressing interleukins have been employed to enhance the anticancer immune surveillance by activating cytotoxic lymphocytes and NK cells (96). Moreover, MSCs expressing interleukin (IL)-12 have been indicated to hinder the metastasis into the lymph nodes and other internal organs as well as promoted tumor cell apoptosis in animal models with pre-established metastases of melanoma, breast, and hepatoma tumor cells (98). IL-12 expressing MSCs were observed to have antitumor functions in mice with cervical tumors (99) and renal cell carcinomas (100). These studies disclosed sustained expression of interferon (IFN)-γ and IL-12 in serum and tumor microenvironment. Additionally, umbilical cord blood-derived MSCs were successfully used as systems to deliver IL-12 to contribute in the malignant glioma therapy (101). As such, the transplantation of MSCs secreting IL-18 was previously indicated to increase T cell infiltration and long-term antitumor responses in mice with non-invasive and invasive gliomas (102). On the other hand, umbilical cord blood-derived MSCs were manipulated to express IL-21 that demonstrated therapeutic efficacy in mice with ovarian cancer xenografts (103). Moreover, the efficacy of IL-24 expressing MSCs as a therapeutic cytokine delivery tool for lung cancer was assessed. Human umbilical cord-derived MSCs modulated to deliver IL-24 inhibited the exacerbation of lung cancer cells by stimulation of apoptosis and arrest of cell cycle (104). Furthermore, this survey demonstrated that injection of MSCs secreting IL-24 repressed xenograft tumor growth and showed anti-angiogenic effects both *in vitro* and *in vivo* evaluations (104). These observations suggest that employing MSCs as vehicles to deliver interleukin have promising effects in treatment of various cancers.

### Interferons

Interferon (IFN)-β has shown anti-proliferative and pro-apoptotic effects on tumor cells (104–106). Therapeutic efficacy of IFN-β has been limited because of toxicity related to systemic administration. To resolve this issue, human MSCs have been engineered to express IFN-β, hence these cells carried out targeted delivery to treat metastatic melanoma and breast models (107, 108), lung metastasis (109, 110), and gliomas (45). Intraperitoneal injection of IFN-β expressing MSCs in mice bearing ovarian xenografts led to complete destruction of tumors in 70% of treated mice (111). These observations validated the potential of MSCs to be employed as targeted delivery vehicle for the production of IFN-β inside the tumor tissue. Furthermore, the intravenous injection of MSCs expressing IFN-β significantly decreased prostate tumor weight and promoted animal survival in comparison to controls (112). Amniotic fluid (AF)-derived MSCs were studied to deliver the IFN-β to the involved site of neoplasia in a bladder tumor model. This investigation indicated that tumor growth was limited and survival of mice was prolonged following administration of AF-derived MSCs secreting IFN-β (113). In previous studies, human adult MSCs engineered for expressing INF-β demonstrated *in vivo* efficacy of these cells to treat the solid melanomas in nude mice (107). The antitumor effects of engineered MSCs expressing INF-β in therapy of central nervous system (CNS) tumors were displayed by evaluating if human MSC could be efficient in tracking the murine brain tumors when administered intravenously (45). Through engineering the MSCs to secrete INF-β, this strategy could be carried out to elicit antitumor effects, as administration of human MSCs secreting INF-β led to enhanced murine survival. Moreover, the efficacy of MSCs secreting IFN-β in a model of prostate cancer and lung metastasis was investigated (110). Targeted colonization of MSCs secreting IFN-β was observed at sites of lung tumors with pulmonary metastases, and this led to inhibition of tumor growth. The antitumor effects of IFN-α, which shows multifunctional regulatory effects, have also been studied. Oftentimes, IFN-α is utilized as a supplementary therapy to destroy the micrometastatic deposits in cases with high risk of systemic recurrence after therapy with other strategies (114, 115). IFN-α expressing MSCs can be efficiently targeted inside the tumor tissue in mice model of plasmacytoma. On the other side, administration of MSCs engineered to express IFN-α through subcutaneous way significantly limited tumor growth *in vivo* and increased the survival of the mice by triggering apoptosis in tumor cells and by diminishing the growth of tumor vessels (115, 116). Moreover, the systemic administration of MSCs expressing IFN-α decreased tumor cell growth and considerably increased the survival rate because of intensified tumor cell apoptosis and decreased development of tumor blood vessels in a mouse model of metastatic melanoma (108, 115).

### Pro-apoptotic Proteins

TRAIL is known as an endogenous member of the TNF ligand family that ligates to its death domain, which bears receptors for Dr4 and Dr5 and triggers apoptosis by activation of caspases, particularly in cancer cells without effect on other cell types (117). Studies have indicated the therapeutic effects of engineered MSCs to express TRAIL in both cell lines or animal models of tumors, such as gliomas (118–120), colorectal carcinoma (121), and lung, breast, squamous, and cervical cancers (122). These investigations disclosed the favorable TRAIL effects in stimulation of apoptosis and a further reduction of tumor cell survival. Studies also indicated that adipose tissue-derived MSCs engineered to express TRAIL targeted several tumor cell lines of human cervical carcinoma, colon cancer, and pancreatic cancer. Following administration into mice, TRAIL expressing adipose tissue-derived MSCs migrated to tumors and triggered apoptosis in tumor cells. Moreover, this targeted delivery resulted in no significant toxicities to normal cells (123). Similarly, adipose tissue-derived MSCs engineered to express TRAIL demonstrated anti-myeloma activities and significantly triggered *in vitro* killing of myeloma cell (124). TRAIL is a type II membrane protein, which needs enzymatic cleavage from its cell membrane anchoring site upon releasing into the microenvironment. In order to eliminate this cleavage step, attempts are on the way to re-design the TRAIL protein in order to achieve TRAIL-secreting cells with direct paracrine function. To engineer the secretable compound of TRAIL, researchers fused the extracellular domain of TRAIL and the extracellular domain of the hFlt3 ligand (125). This re-designed compound, namely S-TRAIL, is efficiently secreted into the tumor microenvironment and indicated excessive cytotoxic effects on tumor cells in comparison to the traditional TRAIL form (126, 127). Investigations disclosed that human MSCs are resistant toward the apoptosis mediated by TRAIL. The cell killing mechanism of MSCs engineered to express S-TRAIL is to trigger caspase-mediated apoptosis in glioma cell lines and glioblastoma stem cells (GBSC) *in vitro* (128).

### Antagonists of Growth Factors

Antagonists of growth factors are beneficial choices for suppressing tumor development. Among the agents that inhibit the function of growth factors, there are a limited number of compounds that can be expressed in stem cells and released in the extracellular microenvironment. As an antagonist of hepatocyte growth factor (HGF) (129), NK4 is a strong suppressor of tumor growth as well as angiogenesis (130, 131). NK4 expressing MSCs has been evaluated in mice models with lung metastases (132). When NK4 expressing MSCs was systemically administered to mice, it localized to the sites of lung metastatic tumor and efficiently suppressed tumor progression/metastases in the lung. Inhibition of lymphangiogenesis and angiogenesis in the tumor tissues has been considered as the mechanism of anti-metastatic action of NK4-expressing MSCs.

## Angiogenesis Perspectives For Cancer Therapy

### Process of Angiogenesis

During embryonic procedures, formation of new blood vessels from pre-existing vasculature is known as a critical event resulting in formation of constant vasculature consisting of endothelial cells (ECs), mural cells, and basement membrane in adults (133). ECs are the most inner layer of vessel walls and are in direct interaction with mural cells along the length of the vessels. The basement membrane is a thin layer that coats the entire length of ECs (134). Vasculogenesis in normal physiological conditions is very stable and is rarely seen in adult individuals. However, adults may develop angiogenesis in cyclical growth of vessels in the ovarian corpus luteum as well as during pregnancy (135). Angiogenesis is defined to be an important event in pathological settings like tissue repair during wound healing as well as during tumor growth (136). Nonetheless, in comparison to normal vessels, the structure and components of tumor vessels are distinct (137). Tumor vessels are disorganized and mazy and do not possess the hierarchy of arterioles, capillaries, and venules. Because the ECs are not in immediate contact with mural cells and are connected to basement membrane loosely in tumor constructions, they are leakier. Overexpression of PlGF, CD109, CD137, and CD276 have been identified in ECs of tumor tissues in comparison to ECs of non-tumor tissues. Although numerous studies have investigated the cellular and molecular features of ECs in tumors, the source of these cells remains controversial in tumors (138). A worthwhile amount of data in certain models recognized endothelial progenitor cells (EPCs) to be directly contributing to tumor angiogenesis (138). Regardless of cellular origin of vasculature in tumors, stimulation and initiation of angiogenesis need a shift to activation and upmodulation of angiogenesis stimulators alongside with suppression of angiogenesis inhibitors. Among the most important stimulatory factors of angiogenesis are MMPs, vascular endothelial growth factor-A (VEGF-A), placenta growth factor (PlGF), fibroblast growth factor (FGF), and hepatocyte growth factor (HGF) (139, 140). On the other side, IL-12, thrombospondins (THSBs), endostatin, and angiostatin are important endogenous inhibitors of angiogenesis (141). In consideration of all, it seems that angiogenesis plays an important role in physiological homeostasis as well as in tumor growth and development.

### Inhibition of the VEGF Pathway as a Major Approach of Anti-angiogenesis Tumor Therapy

With respect of the involvement of angiogenesis in tumor progression and expansion, targeting tumor angiogenesis procedures as a therapeutic tool has long been a putative approach. In fact, suppression of growth factors/signaling pathways required for growth and progression of ECs is considered as a practical strategy to inhibit tumor vasculogenesis (136, 142). Vascular endothelial growth factor (VEGF) has been established as a key modulator of angiogenesis in both physiological and pathological conditions (143). Given the genetic background, a VEGF allele loss, leading to angiogenesis defects, has been observed to be the cause of death during embryonic life (143). Furthermore, VEGF-null embryonic stem cells were shown to be unable to construct teratoma in the recipient upon administration in the testis capsule, implicating the important role of VEGF and thereby angiogenesis in tumor growth and expansion. Among many factors (144), hypoxia-inducible factor (HIF-1a), which binds to VEGF promoter, is one of the major regulators of VEGF expression (145). A hypoxia-conducive environment in different areas of tumors leads to overexpression of VEGF in tumor microenvironment, implicating on accelerated proliferation of tumor cells and faulty blood flow (146). On the other hand, VEGF is highly present in numerous human tumor microenvironments (147), whereas VEGFR1, VEGFR2, and VEGFR3 are overexpressed in tumor associated ECs (148).

To further shed light on the role of VEGF in tumor angiogenesis and devise a therapeutic approach, human tumor-bearing mice were treated with an anti-VEGF neutralizing monoclonal antibody, which significantly suppressed the tumor growth in the mice (149). Bevacizumab, a humanized variant of a VEGF neutralizing monoclonal antibody (150), was approved by the FDA (Food & Drug Administration) in 2003 as a first anti-angiogenic factor as a supplement to therapy with standard of care (SOC) in patients with metastatic colorectal cancer (151). Moreover, bevacizumab was then confirmed to have beneficial effects in treatment of patients with non-small-cell lung cancer (152) and metastatic breast cancer (153). After these prosperous observations, a number of VEGF pathway inhibitory agents have been under different stages of clinical trials, which subject either VEGF or its receptor. VEGF-TrapR1R2, which is a chimeric soluble receptor of VEGF, harbors functional compartments of VEGFR1 and VEGFR2 (154), which have the potential to bind to and thereby neutralize circulating VEGF. Furthermore, VEGF-TrapR1R2 has indicated to be surpassed in anti-tumor properties and function in comparison to other VEGF receptor blockers such as DC101, as observed through preclinical models. On the other hand, inhibition of the VEGF pathway by means of VEGF receptor blockades also suppresses the tumor development (155, 156). As an instance, receptor tyrosine kinase inhibitors (RTKIs) have been evaluated, which is receptor tyrosine kinases inhibitor and targets VEGF and other molecules involved in VEGF signaling pathways. Among the most clinically promising RTKIs are linifanib (157, 158), cabozantinib (159), axitinib (160), tivozatinib (161), vendatanib (162), sunitinib (163), pazopanib (164), and sorafenib (165). While clinical measurements imply that there are beneficial effects of these compounds on tumor treatment, the precise mechanism of action of these anti-angiogenic agents in cancer therapy is still obscure. Angiogenesis inhibitors targeting the VEGF pathway have been indicated in preclinical models to repress tumor development through inhibition of tumor angiogenesis. Nonetheless, other studies show that anti-angiogenesis agents may function through vascular normalization, in which these agents target the non-functional vessels in tumors, leading to diminished blood flow, enhanced tumor oxygenation, and better delivery of cytotoxic drugs to the tumor tissue (166). It should be noted that the results of preclinical models have been in contrast with those of clinical trials with respect to VEGF inhibitors. Clinical trials have indicated different results between the efficacy of monoclonal antibodies in comparison to small anti-angiogenesis inhibitors. Bevacizumab in combination with SOC indicated favorable results in patients with metastatic breast cancer (153), colorectal cancer (151), and non-small cell lung cancer (152). It can be concluded that development of VEGF-related and anti-angiogenic compounds provided a promising therapeutic tool for cancer disorders and opened new horizons within this context. In addition to VEGF inhibitors, other classes of anti-angiogenesis agents, namely vascular disrupting agents (VDAs), have been evaluated. VDAs function in suppressing the tumor progression through triggering vascular collapse, resulting in hypoxia and thereby necrosis of tumor cells (167). As a VDA, ASA404, which is a flavonoid compound, induced apoptosis in tumor ECs, leading to a blockade of blood flow in tumor tissues. Currently, the ASA404 is under clinical evaluations in patients with non-small cell lung cancer (168).

### Inhibition of Signal Transduction by Targeting Receptor Tyrosine Kinases

Angiogenesis is mediated by ligation of growth factors to their receptors, which results in signal transduction through corresponding receptor tyrosine kinases (RTKs) (169). Three subfamilies of 14 RTK, namely Ang/Tie-2, VEGF/VEGFR-2, and Ephrin B2/EphB4 are particularly overexpressed in the endothelial cells. These three RTKs are essentially involved in the development of both vasculogenesis and angiogenesis (170). VEGF binding to VEGFR-2 induces signal transduction that eventuates in survival and proliferation of endothelial cells. Vessel stabilization and maturation as well as remodeling of vessel structure have been associated with angiogenic growth factors (Ang) and their tyrosine kinase receptor (Tie-2) (171). Moreover, Ephrin receptor tyrosine kinase (EphB4) and its transmembrane-type Ephrin B2 ligand play a role in vasculogenesis (172). The small molecules inhibiting the angiogenesis were (2-(3,4-Dihydroxyphenyl)-6,7-dimethylquinoxaline-HCl and (E)-3-(3,5-Diisopropyl-4-hydroxyphenyl)-2-[(3-phenyl-n-propyl) aminocarbonyl] acryl-onitrile), which were first identified in 1996. These molecules inhibited VEGF-dependent VEGF receptor-2 phosphorylation, and therefore hindered endothelial cell mitogenesis and blood vessel development (173). Targeting these RTKs is considered as a potentially therapeutic approach via designing novel anti-angiogenesis agents. For example, sorafenib, which is a multikinase inhibitor, interrupts tumor growth via antiproliferative and antiangiogenic effects. Sorafenib, which inhibits PTKs, has demonstrated antitumor activity in phase 3 trials in patients with advanced renal cell carcinoma and hepatocellular carcinoma (174). Moreover, a phase 3 trial of sunitinib, which is a RTK inhibitor, in patients with gastrointestinal stromal tumors has demonstrated promising outcomes (175).

### MSCs and Anti-angiogenic Cancer Therapy

Through angiogenesis, tumors provide a way to continue to growth and progression (5). One of the approaches to employ anti-angiogenetic agents to limit the tumor growth and metastasis is to use MSCs as vehicles to deliver such agents. These manipulated MSCs demonstrated a tropism to cancer tissue and may deliver antiangiogenic compounds without adverse side effects (176). Moreover, BM-MSCs were indicated to be able to modulate the expression profile of chemokine genes (including CXCL10, CXCL3, CXCL6, and CCL-2) that play a role in angiogenesis (177). Nonetheless, prolonged systemic delivery of anti-angiogenic agents is related with toxicity, as well as poor blood supply, which decreases the delivery of chemotherapeutic drug agents to tumor cells (178). It has been identified that tumor-mediated angiogenesis stems from an aberrant regulation of both pro-angiogenic and antiangiogenic agents as well as via growth factors of the tumor microenvironment (6, 179). Endostatin, which is an important endogenous inhibitor of tumor angiogenesis, has been widely utilized in antiangiogenic therapy to treat various cancers (180). Human placenta-derived MSCs were engineered to deliver endostatin through adenoviral transduction and were then administered into nude mice. These engineered MSCs expressing the human endostatin exhibited homing to the tumor tissue and considerably diminished the tumor size, with no remarkable systemic toxic side effects. These beneficial effects in tumor therapy were due to enhanced tumor cell apoptosis, inhibited neovascularization, and hence diminished tumor cell proliferation and expansion (180). In addition, a phase II clinical study demonstrated that the delivery of anti-angiogenic agents normalized the abnormal structure and defective function of the blood vessels, which led to significantly reduced tumor-associated vasogenic brain edema (181). The vessel normalization was attributed to reduced vessel diameter and permeability (182, 183) as well as the increased mural cell coverage of the small vasculature (184). Studies show that MSCs possess the ability to home to tumor vasculature following intratumoral administration, suggesting favorable characteristics for targeted delivery of anti-angiogenic agents, especially in vascularized cancers (185).

### Angiogenesis Inhibition; MSC or Other Antiangiogenic Factors

As discussed above, several antiangiogenic compounds, such as monoclonal antibodies against VEGF or VEGFR, have been addressed in cancer anti-angiogenic therapy. On the other side, MSCs have also been reported to modulate the tumor microenvironment to suppress angiogenesis or neovascularization. Each of these approaches has its pros and cons. The targeted delivery of MSCs provides a favorable characteristic in the treatment of solid tumors. Furthermore, MSCs can be engendered to deliver anti-angiogenetic agents, supporting an efficient MSC to treat angiogenesis. Nonetheless, MSCs may manifest with some unwanted properties. Among the critical concerns that requires precise evaluation before MSCs' clinical application in humans, is the safety issue. Although there are several clinical reports and current clinical trials, little has been revealed with respect to the long-term safety of MSCs in humans. Furthermore, the potential profibrogenic capacity of MSCs, as indicated in liver fibrosis triggered through human BM-MSCs (186), confers another limitation in their therapeutic application. Another issue is the heterogeneity of MSCs as a source for their application in clinical settings. MSCs are a heterogeneous pool of various cell populations which are characterized via cell surface molecules as well as by their ability to differentiate into several lineages, such as chondrocyte, osteoclast, or adipocyte. Heterogeneity of MSCs (obtained from donors as a cell source) may stem from aging, age, sex, genetics, and epigenetic factors (187). Another problem with application of MSCs to treat cancers with suppressing angiogenesis is that MSCs may confer a potential to develop angiogenesis, a characteristic in reverse to desirable purpose (188). Despite such obstacles, researchers have been surveying to increase the efficacy of MSC-based cancer therapy with respect to suppression of angiogenesis.

## Putative Concept in Application of Anti-angiogenic Inhibitors and MSC Delivery Machine

Estrogen is the primary female sex hormone which regulates the growth, differentiation, and physiology of the reproductive process through binding to the estrogen receptor (ER). Estrogen plays a role in several tissues, including liver, bone, brain, and the cardiovascular system. Since estrogen's ligation to ER indicated diverse outcomes, disclosing the basis of ER functions at the molecular level has mainly been of interest in the aim of therapeutic interventions (189). Current evidence shows that estrogen induces angiogenesis by acting either directly on endothelial cells and/or indirectly on other endometrial cell types through several potential promoters. Estrogen stimulates VEGF expression in human uterine stromal cells (190, 191). Studies show that ER is a potentially favorable target to devise drugs for the treatment and prevention of the progression of breast cancer (192). Estrogen binding to ER can lead to enhanced proliferation of target cells; hence endocrine cancer therapy aims to block the interaction of estrogen with the ER. This aim is achieved through either blocking the production of estrogen or suppressing the conversion of steroidal precursors to estrogen. Selective estrogen receptor modulators (SERMs), such as tamoxifen and raloxifene, which are competitive inhibitors of estrogen, directly target ER. Manipulating the estrogen pathway is considered as the most effective therapy, with less toxic side effects for the treatment of hormone responsive breast cancers (193).

SERMs' function in select target tissues is like estrogen's but is like an anti-estrogen in other target tissues (194). The ideal SERM should act like an estrogen agonist in bone, liver, the cardiovascular system, and brain and an estrogen antagonist in the uterus and breast. On the other hand, the activity of tamoxifen relies on circulating levels of E2, which are in low levels in postmenopausal women and in high levels in premenopausal women. Tamoxifen treatment reduces bone density of premenopausal women but increases that of postmenopausal women (195). Furthermore, estrogenic activity of tamoxifen has been observed in the uterus, leading to high incidence of endometrial cancer in postmenopausal women (196). In prostate cancer, involvement of ER has been implicated. Moreover, overexpression of ERβ has been reported in the prostate (197). Within the prostate, ERβ is primarily found in the epithelium, while ERα is localized in the stroma (198). The prostate gland highly expresses ERβ, suggesting its susceptibility to the effects of estrogens (199). Application of estrogen therapy has been promising in ovarian cancers (200). The Women's Health Initiative (WHI) trial has provided evidence on the role of estrogen in colon carcinogenesis (201).

Together, given the role of estrogen and ERs in angiogenesis as well as promising therapeutic effects of inhibition of this pathway in several cancers, MSC application as a targeted delivery vehicle is suggested. Moreover, monoclonal antibodies against ERs can be employed to be directly present in the tumor microenvironment to inhibit angiogenesis. Additionally, administration of SERMs has indicated side effects in untargeted tissues. This way, estrogen/ER pathways are optimally manipulated to elicit better outcomes. This hypothesis is premature, which needs to be addressed in animal models and clinical trials. On the other side, progesterone also inhibits endothelial cell proliferation *in vitro* and arrests the cell cycle in the G0/G1 phase in human dermal endothelial cells (202). Progesterone receptors have been found in human endometrial endothelial cells and, when interacted with progesterone, suppressed VEGF-induced endothelial cell proliferation (203). Targeting the progesterone signaling pathway through engineered MSCs seems to be a plausible therapeutic tool for efficient cancer treatment, requiring extensive investigations.

The corpus luteum is characterized as a temporary endocrine structure in ovaries, which plays an important role in successful pregnancy in mammals. Upon rupture of follicle following ovulation, the corpus luteum is made from the debris and then is modulated through differentiation and growth. Studies indicate that the fast growth and development of the corpus luteum is contingent upon the angiogenesis process. A number of endogenous stimulatory and inhibitory factors are expressed in the corpus luteum to balance the luteal angiogenesis process (204). Pigment epithelium–derived factor (PEDF) is a physiological inhibitor of angiogenesis (205), which is mainly secreted by granulosa cells. Thrombospondins 1 and 2 are also antiangiogenic factors. Thrombospondin 1 binds to fibroblast growth factor 2 (FGF2) and then prevents its proangiogenic functions (206). Angiogenesis regulation mediated by thrombospondins is important for the diminishing of corpus luteum. Studies show that thrombospondins 1 expression was upregulated during luteolysis in an animal model (207). Additionally, prostaglandin PGF2a stimulates the overexpression of thrombospondins 1 and 2 as well as their receptor CD36 in the bovine corpus luteum. Inhibition of angiogenesis in the corpus luteum is mediated in response to the mentioned molecules in the presence of FGF2. However, inhibition was also seen in lack of FGF2 (208). These molecules play roles in inhibition of physiological angiogenesis in the corpus luteum, which might open new horizons in anti-angiogenesis therapy of cancer in the future, possibly by application of MSCs as vehicles to deliver these anti-angiogenetic mediators into the tumor tissue.

Recently, it has been established that exosomes derived from MSCs can deliver microRNAs (miRs) to target cells and, therefore, impress the angiogenesis. It was observed that incubation of MSCs with the exosome secretion blocker GW4869 decreased the levels of pro-angiomiRs in the MSC-derived medium (209). This observation led to the implication of the MSC-derived exosomes in inhibition of angiogenesis. In addition, an anti-tumor role of intra-tumoral injection of menstrual stem cell-derived exosomes was associated with an inhibitory impression on angiogenesis in oral squamous cell carcinoma (210). Moreover, MSCs were observed to suppress the angiogenesis potential of HUVEC through overexpressing miR-211, which downregulated the expression of Prox1 (211). Additionally, it was reported that MSC-derived exosomes, which were enriched with miR-16 (a miRNA targeting VEGF), could downregulate the expression of VEGF in tumor cells, resulting in suppression of angiogenesis both *in vitro* and *in vivo* (212). As a consequence, delivering exosomes expressing miRNAs (targeting angiogenetic molecules) by MSCs may also confer a potential tool in the suppression of angiogenesis.

## Clinical Trials and MSCs-Based Therapy of Cancer

There is a paucity of registered clinical trials with the aim of cancer therapy via exertion of MSCs. The first clinical trial (a phase I/II clinical trial), namely Treatment of advanced gastrointestinal tumors with genetically modified autologous mesenchymal stromal cells (TREAT-ME1), applied MSC delivery of herpes simplex virus thymidine kinase (HSV-TK), which had prosperous results (213). There are two clinical trials focusing on ovarian cancer via application of MSCs. A phase I/II trial by Mayo Clinic purposed to recognize the adverse effects and well-suited dose of MSCs transfected with oncolytic measles virus encoding NIS (MV-NIS) and to evaluate its efficacy on patients with ovarian cancer. The University of Texas M.D. Anderson Cancer Center supported a phase I clinical trial that used human MSCs transfected with IFN-β to assess its safety and to identify the well-tolerable dose of such MSCs in treating ovarian cancer patients. In another phase I clinical test, aiming to investigate the safety and cancer-homing potential of MSCs, allogeneic BM-MSCs were administered in patients with prostate cancer. Nonetheless, the study revealed that MSCs could not efficiently home in on tumor sites to implement therapeutic functions (214). These studies have attempted to evaluate the potential of MSCs in treating various cancers; however, the results seem to be partially ineffective. That notwithstanding, further studies are required focusing on the efficacy and safety of such a therapeutic system through transforming the observations from bench (preclinical studies) toward the bed side (clinical uses).

## Conclusion and Future Challenges and Perspectives

**Figure 3:**
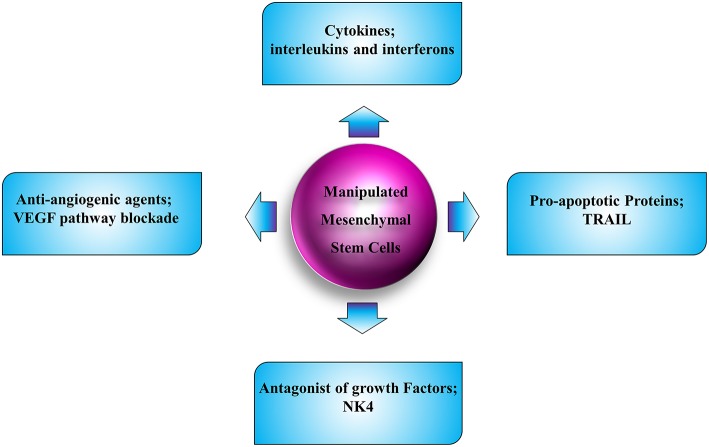
Schematic illustration of strategies for tumor therapy with employment of MSC.

MSCs are able to preferentially infiltrate into the solid and disseminated tumor tissues and interact with different cells in the tumor microenvironment. Additionally, they are easily available, non-immunogenic, and simply manipulated *in vitro* without needing to be immortalized; hence they are the most appropriate candidates for cell-based therapies in human cancers (Figure 3). Despite bone marrow, adipose, umbilical cord blood, and local tissues are suitable sources of autologous and allogeneic MSCs; however, their biological regulation and kinetics need to be further elucidated. A number of preclinical investigations in which MSCs have been utilized as vehicles to express a single therapeutic molecule for localized delivery have been explored in different cancer types. Although anti-angiogenic therapy has increased survival of cancer patients, the pooled results indicated that the overall survival improvement was limited (215, 216). The limitations of utilization of anti-angiogenesis therapy are due to several reasons. First, because the angiogenesis is a complicated procedure with involvement of a network of mechanisms, the tumor microenvironment plays a pivotal role in mediating resistance in treatment with angiogenesis inhibitors (217). Second, studies implicate that angiogenesis inhibitors culminating in vascular regression could elevate intratumoral hypoxia. This increased hypoxia results in radio-resistance, chemo-resistance and anti-angiogenesis resistance, since most of the pro-angiogenic factors with tumor aggression effect are mainly produced and released by tumor cells within a hypoxic environment (218, 219). Third, the systematic decrease of tumor vasculature and blood flow leads to reduced delivery of chemotherapeutic agents. These complications may result in tumor metastasis and negatively affect the clinical employment of angiogenesis inhibitors. Although our understandings of the mechanobiology of MSCs have improved greatly over the course of the past few years, we are still on the initiatory steps toward being armed with satisfactory knowledge of these cells and exertion of these cells in clinic. The gap here could be filled by novel hypotheses in the application of anti-angiogenic factors with less adverse effects and their related pre- and post-modulators. Achieving an optimal anti-angiogenic therapy needs complex solutions. Engineering MSCs to selectively deliver the anti-angiogenic molecules could still be promising. Additionally, exploring novel molecular targets contributes to the angiogenesis inhibitors as favorable cancer therapy agents in the future. Other than that, more clinical trials are mandatory to test for the efficacy and safety of such new strategies.

## Author Contributions

MJ performed the literature search and prepared the draft of the paper. AK drew the figures and read the manuscript critically. SM developed the main idea, designed the work, and read the manuscript critically.

### Conflict of Interest Statement

The authors declare that the research was conducted in the absence of any commercial or financial relationships that could be construed as a potential conflict of interest.
